# Single-Site Mutation Induces Water-Mediated Promiscuity in Lignin Breaking Cytochrome P450_GcoA_

**DOI:** 10.1021/acsomega.2c00524

**Published:** 2022-06-10

**Authors:** Warispreet Singh, Sónia F. G. Santos, Paul James, Gary W. Black, Meilan Huang, Kshatresh Dutta Dubey

**Affiliations:** † Department of Applied Sciences, Northumbria University, Newcastle upon Tyne NE1 8ST, United Kingdom; ‡ Hub for Biotechnology in Build Environment, Newcastle upon Tyne NE1 8ST, United Kingdom; § Department of Chemistry & Chemical Engineering, Queen’s University, Belfast BT9 5AG, United Kingdom; ∥ Department of Chemistry and Centre for Informatics, Shiv Nadar University Delhi NCR, Gautam Buddha Nagar, U.P. 201314, India

## Abstract

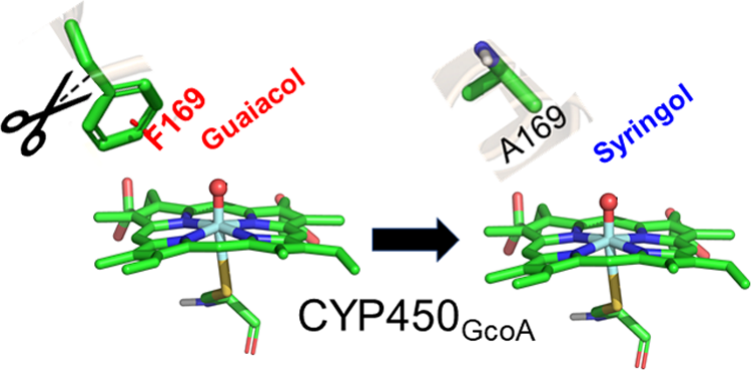

Cytochrome P450_GcoA_ is an enzyme that catalyzes the guaiacol unit of lignin during the lignin breakdown via an aryl-*O*-demethylation reaction. This reaction is intriguing and is of commercial importance for its potential applications in the production of biofuel and plastic from biomass feedstock. Recently, the F169A mutation in P450_GcoA_ elicits a promiscuous activity for syringol while maintaining the native activity for guaiacol. Using comprehensive MD simulations and hybrid QM/MM calculations, we address, herein, the origin of promiscuity in P450_GcoA_ and its relevance to the specific activity toward lignin-derived substrates. Our study shows a crucial role of an aromatic dyad of F169 and F395 by regulating the water access to the catalytic center. The F169A mutation opens a water aqueduct and hence increases the native activity for G-lignin. We show that syringol binds very tightly to the WT enzyme, which blocks the conformational rearrangement needed for the second step of O-demethylation. The F169A creates an extra room favoring the conformational rearrangement in the 3-methoxycatechol (3MC) and second dose of the dioxygen insertion. Therefore, using MD simulations and complemented by thorough QM/MM calculations, our study shows how a single-site mutation rearchitects active site engineering for promiscuous syringol activity.

## 1 Introduction

Cytochrome P450 is nature’s ultimate enzyme that can catalyze a plethora of chemical reactions.^1−5^ It is biological machinery that works in a well-organized catalytic cycle using molecular oxygen, water molecules, and electrons as fuel for the efficient oxidation of several compounds of commercial and medicinal importance.^6−12^ Due to the catalytic versatility of P450 enzymes, it is no surprise that cytochrome P450 is the most widely used scaffold for bioengineering of new catalytic functions.^13−15^ Recently, three members of the CYP450 superfamily, P450_OleT_ from the peroxygenase family,^16,17^ P450_GcoA_ from CYP255A2,^18−20^ and AgcA from the CYP255A1^21^ family, were found to catalyze reactions of potential biofuel importance.^22^ P450_GcoA_, in particular, gained special attention as it catalyzes the downstream degradation of lignin, which can potentially be used to produce a drop-in biofuel.^23^Lignin is ubiquitous in the biosphere, and it alone constitutes 15–30% of lignocellulosic biomass (LB). Moreover, the pulp and paper industry alone produces around 50–60 million tons of lignin each year, which further adds to this enormous renewable carbon feedstock.^24,25^ Lignin biomass, therefore, is an unexploited treasure that provides an excellent source of sustainable aromatic carbon. Hence, understanding the mechanism of downstream lignin breakdown for potential biofuel production could be pivotal for bioengineering of this natural enzyme to enhance biofuel production.

**Scheme 1:**
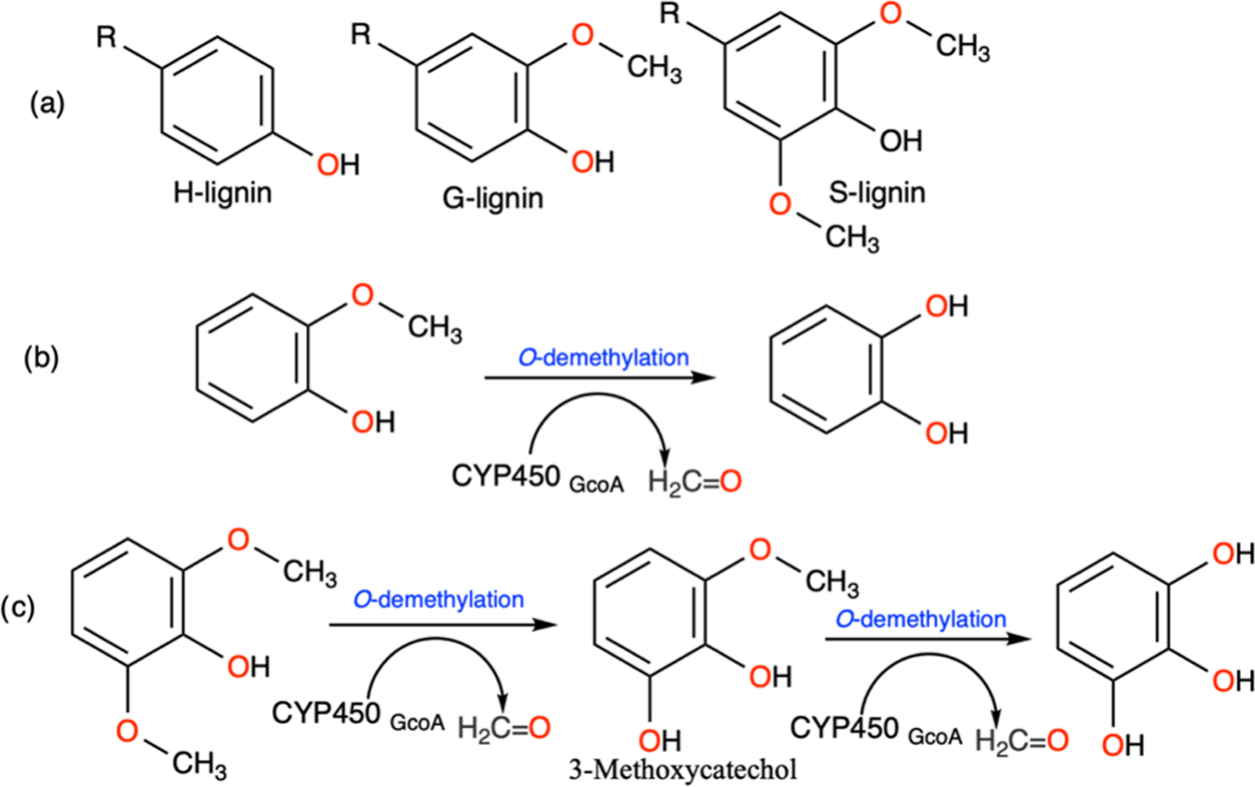
(a) Structure of Primary Monomeric Units of Lignin, (b) Aryl-*O*-demethylation Reaction Catalyzed by the P450 Enzyme of Guaiacol, and (c) Aryl-*O*-demethylation Reaction Catalyzed by the P450 Enzyme of Syringol Syringol is subject to dual demethylation.

**Scheme 2:**
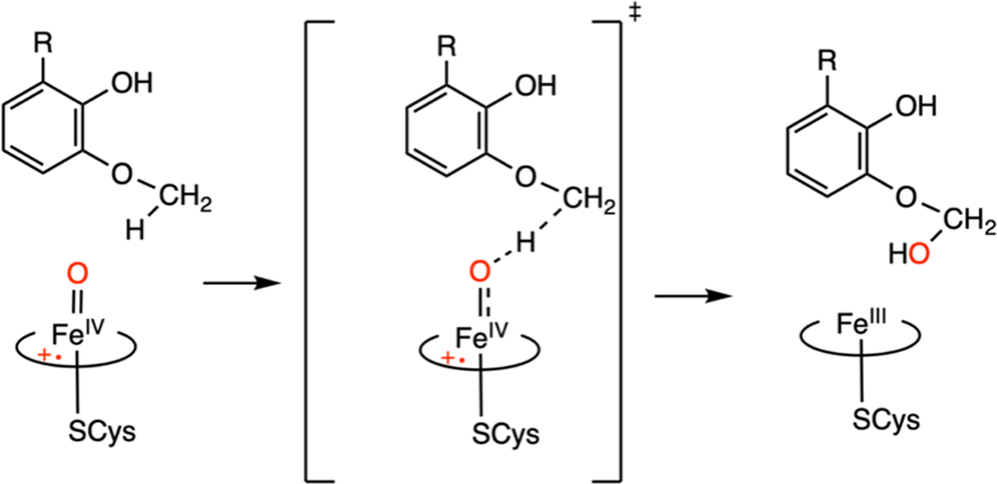
Hydrogen Atom Abstraction by Cpd I from Guaiacol by P450_GcoA_ (R = H, O–CH3, OH).

Chemically, lignin is a recalcitrant polymer that is composed of three alcoholic units: p-coumaryl alcohol (H-lignin), coniferyl alcohol (G-lignin), and synapyl alcohol (S-lignin) (Scheme 1A).^25,26^ The catabolism of G and S units (here represented by guaiacol and syringol, G and S unit monomers derived from lignin) into biomass products is particularly of importance as both are major components of lignocellulose biomass. This catabolic reaction is achieved through aryl-*O*-demethylation (Scheme 1B) via the action of Cpd I (Fe(IV)=O porphyrin radical cation), which is also the main oxidant in P450-catalyzed reactions.^11,12^ The reaction is initiated by the oxo group of Cpd I by abstracting a hydrogen atom from the sp^3^-hybridized C–H bond of the methoxy group of the substrate (Scheme 2). This is followed by the rebound step, where the hydroxyl group is transferred to the substrate to form a hemiacetal product. The previous gas-phase DFT calculations with implicit solvent corrections have identified the hydrogen atom abstraction (HAA) as the rate-limiting step and a barrierless rebound step for both the guaiacol and syringol substrates.^19,20,27,28^

Unfortunately, the native P450_GcoA_ enzyme shows aryl-*O*-demethylase activity toward guaiacol only but not against syringol.^20^ This creates an initial roadblock to achieving greener and sustainable downstream lignin biodegradation through a natural enzymatic process. Very recently, using a site-directed bioengineering approach, Machovina et al. engineered the native P450_GcoA_^19^ by mutating the bulky phenylanaline at 169 position into smaller alanine and achieved promising activity for syringol (which carries an additional methoxy group in comparison to guaiacol), while it increases the native activity by two times toward the transformation of guaiacol as well. The X-ray structure of F169A in a complex with syringol shows that binding of syringol is identical to guaiacol binding in wild-type P450_GcoA_.^19,20^ To explain the experimental findings, the MD simulations on F169A and wild-type P450_GcoA_ in complex with syringol and guaiacol concluded that the phenylalanine residues (F75, F169, and F395) helps to position the substrate in the appropriate orientation for demethylation reaction and the mutation of F169 to alanine creates an extra space that allows for the rotation of the substrate in a favorable orientation for the demethylation of syringol.^19,20^ However, the underlying structure feature that accounts for the promiscuous activity of P450_GcoA_ to accommodate different lignin-derived compounds, is still not largely clear. The previous literature on the wild-type P450_GcoA_ in complex with substrate guaiacol or product catechol induces a partially open and closed conformation of the substrate access channel.^19,20^ However, F169A in complex with guaiacol and syringol showed an increased probability of an open substrate access channel and exposes the active site of P450_GcoA_ to the bulk solvent.^19^ Previous DFT calculations^19,20,27,28^ on the HAA and the rebound step of guaiacol or syringol in complex P450_GcoA_ and its variant F169A neglected the effect of the open substrate access channel, the role of solvent in catalysis and the demethylation of the second methoxy group of syringol.

Here, we investigated the binding and catalytic mechanism of the promiscuity for both syringol and native guaiacol using comprehensive MD simulations to improve sampling (16 replicas × 500 ns; see SI Table S1) and hybrid state-of-the-art QM/MM calculations that take into account the whole protein environment that is essential for enzyme catalysis^11,29^ to address the following mechanistic puzzles: (a) In contrast to guaiacol that does not change the orientation in different P450_GcoA_ variants,^19,20^ why does the enzyme shows prominent alternation in the active site when it is bound with syringol (S-unit)? (b) Whether the water in the F169A mutant plays a role in the spontaneous promiscuity for different lignin-derived compounds? (c) In contrast to guaiacol where the substrate needs a single oxygen insertion to form the final product, the conversion of syringol to pyrogallol (the final product) needs two oxygen insertions. Therefore, how does the second methoxy group of syringol (3-Methoxycatechol) gets demethylated to the final product and whether the second reaction sphere plays a role in the promiscuous activity of the F169A mutant?

In the present study, using extensive MD simulations and hybrid QM/MM calculations, we will show that in addition to active site plasticity, rerouting of the water aqueduct also is critical in the promiscuity activity of the P450_GcoA_ enzyme.

## 2 Methods

### 2.1 Structure Preparation

The X-ray structures of the wild-type CYP P450_GcoA_ and F169A mutant in a complex with different ligands^19,20^ were used as the starting structures for this computational study. (Table S1). The protonation states of the titratable residues were predicted using the H++ server^30^ at pH 7.5 in accordance with the previous literature.^19,20^ The resting state and the compound I state of GcoA_P450_ were modeled using previously published parameters.^31^ The general Amber force field (GAFF)^32^ was used to obtain the parameters for syringol, guaiacol, and 3-methoxycatechol (3MC). The partial charges for these ligands were computed using the Gaussian16 package by performing quantum mechanical (QM) calculations at the HF/6–31G*^33^ level of theory using the RESP (restrained electrostatic potential) method. 3MC was docked into the active site of wild-type CYP P450_GcoA_ and F169A mutant using AutoDock4.2 to obtain the enzyme–ligand complex for subsequent modeling (see the SI for more details).^34^ The Amber ff19SB^35^ force field was used to model the protein molecule, which was then inserted into the TIP3P truncated octahedral water box.^36^ The protein molecule in the simulation box was kept at a distance of 15 Å away from the box edges. The periodic boundary conditions were used in each simulation run. Particle mesh Ewald was used to compute the long-range electrostatic interactions with a cutoff value of 12 Å.

### 2.2 MD Simulations

The Compute Unified Device Architecture (CUDA) version of particle mesh Ewald molecular dynamics (PMEMD) was used to run all of the MD simulations using graphics processing units (GPUs)^38−40^ in Amber20.^37^ The energy minimization was run for 5000 steps using the steepest descent and conjugate gradient method to relax the entire system for subsequent heating and equilibration steps. The system was then heated from 0 to 298.15 K using the Langevin thermostat with a collision frequency of 1 ps^–1^ for 50 ps. All of the atoms except for hydrogen of the solute were restrained with a harmonic potential of 5 kcal mol^–1^ Å^2^. After heating, the entire system was once again energy-minimized for 2000 steps before subjecting to equilibration processes. The equilibration was run at 298.15 K for 50 ps using the NPT ensemble. During the equilibration, all of the solute atoms were held with a weak restraint of 0.1 kcal mol^–1^ Å^2^ and a pressure of 1 bar using a Berendsen barostat. The productive MD simulations for each complex and its replicas were run for 500 ns in the NPT ensemble with a time step of 2 fs. The bonds involving hydrogen atoms in the simulations were constrained using SHAKE. The analysis of the MD trajectories was conducted using the Jupyter notebook.^41^

### 2.3 QM/MM Calculations

Hydrogen atom abstraction (HAA) and the rebound step of wild-type CYP450_GcoA_ and its variants in a complex with different substrates were computed in this study. Prior to the quantum mechanics/molecular mechanics (QM/MM) geometry optimization, the MD snapshots were energy-minimized for 2500 steps using the steepest descend and conjugate gradient algorithms. The water shell of 4 Å surrounding the whole protein was retained while the excess solvent was discarded for the QM/MM calculations. The region of the protein and solvent molecule within 10 Å of Cpd I was treated as an active region and was allowed to optimize freely, while the remainder of the protein was kept frozen. The QM/MM calculations were implemented in Chemshell3.7,^42^ where ORCA4.2.0^43,44^ and DL_POLY^45^ were used to perform the QM and MM calculations, respectively, using an electronic embedding scheme.^46^ The QM atoms consist of Cpd I, ligands, and the cysteine residue truncated at Cβ positions (SIScheme 1). A larger QM region consisting of extra three F75, F169, and F395 was also used to perform QM/MM calculations for the HAA step (SIScheme 1). DFT using the UB3LYP functional^47,48^ with D3 dispersion correction and BJ damping^49^ was used to run QM calculations. The def2-SVP def2/J auxiliary basis sets were used for all of the atoms along with RIJCOSX approximation. The keywords such as TightSCF, slowconv, Grid4, and GridX4 were also used in QM calculations. The final energies were also computed at the UB3LYP-D3BJ/def2-TZVPP level. The QM/MM potential energy scan (PES) was run for the HAA step by decreasing the distance (0.1 Å) between the oxygen atom of Fe(IV)=O in Cpd I and the hydrogen atom to be abstracted from the substrate. The transition states (TSs) were fully optimized using the dimer method of Chemshell3.7^42^ and validated by the presence of one unique imaginary frequency. The normal mode of the imaginary frequency corresponds to the transition of the hydrogen atom from the C–H bond of O–Me to be demethylated to the oxygen atom of Fe(IV)=O of Cpd I. The HAA and the rebound step were studied for all of the variants at *S* = 1/2.

## 3 Results and Discussion

### 3.1 Water Channel in P450GcoA F169A in a Complex with Guaiacol Disclosed by MD Simulations

**Figure 1:**
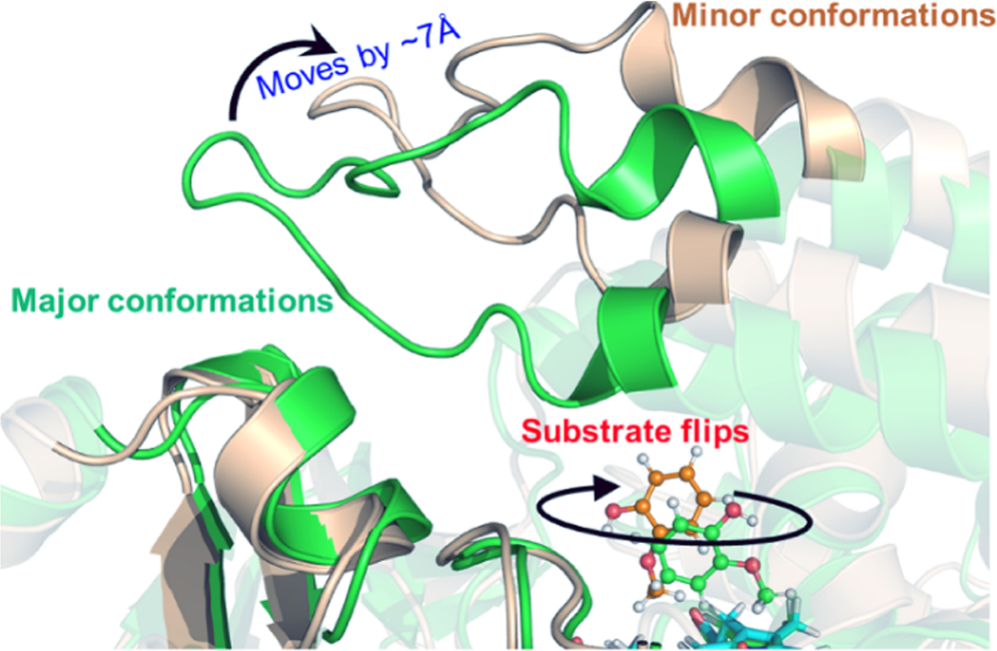
Two different conformations based on the substrate access channel of the WT P450_GcoA_ enzyme in a complex with guaiacol. The protein and guaiacol structures are shown in ribbon and ball-and-stick representation, respectively. The green color shows the major conformation, while the light gold color shows the minor conformations.

**Figure 2:**
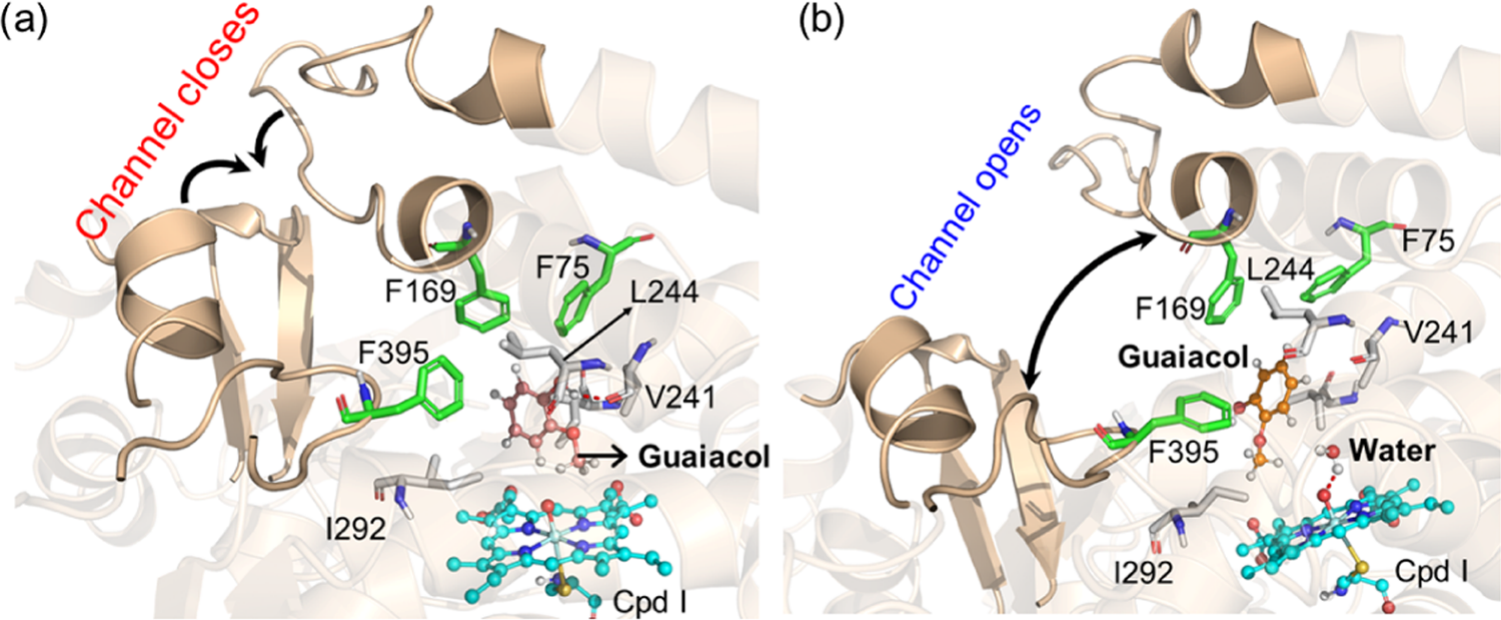
Substrate access channel of the WT P450_GcoA_ enzyme. (a) Closing and (b) opening of the substrate access channel. The arrows in the figures show the movement of the structure *during* the closing and opening of the channel.

The MD simulations of the WT P450_GcoA_ enzyme in the presence of guaiacol show two different conformations based on the substrate access channel of the enzyme (see Figure 1): one is open and the other is the closed conformation. In the majority of the MD trajectories (SI Figures S1 and S2), the substrate access channel of the enzyme maintains a closed conformation (Figure 2a). This is in accordance with the MD simulations performed by Mallinson et al., where WT P450_GcoA_ in a complex with guaiacol and catechol remains in a closed conformation and partially visits the open conformation.^20^

In this conformation (i.e., major conformation), substrate guaiacol was oriented in a vertical position relative to the porphyrin ring of Cpd I similar to the crystallographic structure and previous computational studies.^20^ We found that the substrate-binding site is mainly occupied by an aromatic triad composed of F75, F169, and F395 and several additional hydrophobic residues such as I81, V241, L244, and I292. The role of the aromatic triad has been proposed as crucial for substrate orientation.^18−20^ interestingly, during simulations, none of the phenylalanine residues of the aromatic triad (F75, F169, and F395) form direct contact with the substrate, and therefore, they maintain the rigidity of the active site to keep the substrate in a proper orientation.

Furthermore, the backbone oxygen of V241 forms a strong and persistent hydrogen bond with the hydroxy group of guaiacol, which places the methoxy group in an optimal orientation for hydroxylation via the heme center and is consistent with the previous literature.^19,20^ The hydrophobic triad of F75, F169, and F395 was in a locked position in the substrate access channel and does not show flexibility during entire MD simulations. Interestingly, F169 and F395 act as gates of a doorway that regulates water inflow to the active site. In the majority of MD trajectories, this doorway remains closed due to the locked-in position of F169 and F395 residues, blocking the access of water molecules to the substrate/heme center (Figure 2a).

In the minor conformational basin, the enzyme–substrate complex shows an open conformation of the substrate access channel. In this conformation, F169 and F395 move apart to open the water doorway and allow the water influx (Figure 2b). The simulations performed by Mallinson et al. also indicate the movement of F169 and F395 due to open conformation; however, there was no influx of water in the active site.^20^ The increased water influx exerts a pressure that changes guaiacol orientations, which, in turn, pushes the methoxy group away from the oxygen atom of the Fe (IV)=O complex. The different water accumulations in major and minor conformations are substantiated by radial distribution functions shown in SI Figure S3. In summary, an array of triad residues F169 and F395 in association with water inflow regulates the substrate orientation and hence the guaiacol activity.

**Figure 3:**
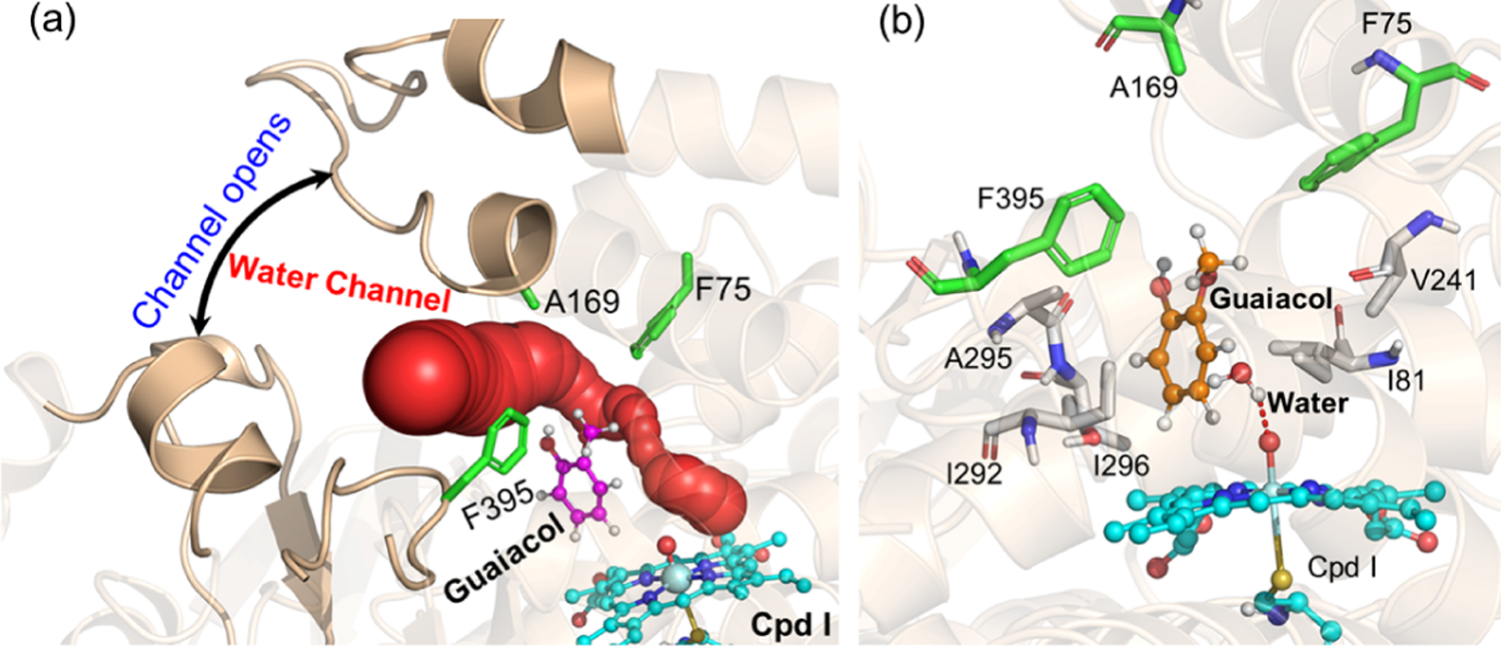
Water channel formation in the F169A P450_GcoA_ enzyme in a complex with guaiacol. (a) Water tunnel formed due to substrate access channel opening and (b) flexibility of guaiacol in the active site and water interactions with Cpd I.

To further elucidate the critical role of the residue at position 169 in mediating water gating, we simulated the F169A mutant of the P450_GcoA_ complex. Interestingly, we found that changing the bulky phenylanaline into a small residue alanine F → A mutation opens the water gateway, and a persistent water channel is stretched from the surface to the heme-binding site (Figure 3a). This water chain might have further implications on enhanced native activity and promiscuity in the F169A variant as observed compared to the WT (Figure 3).

### 3.2 Mechanism of Aryl-*O*-demethylation of Guaiacol by the WT and F169A Mutant

**Figure 4:**
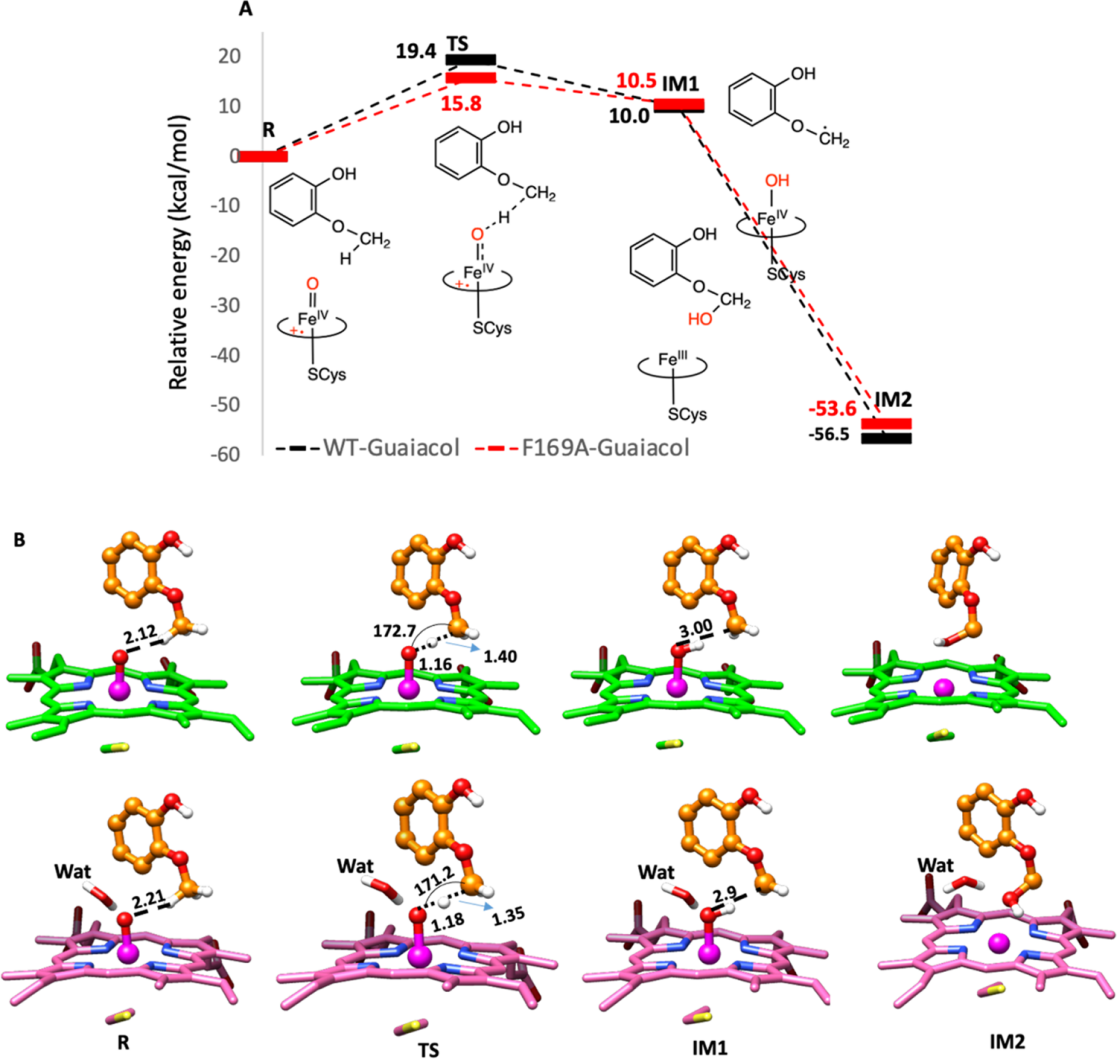
Oxidation of guaiacol in the active site of the WT and F169A P450_GcoA_ enzyme. (A) QM/MM reaction profile was computed using the UB3LYP functional with D3BJ dispersion correction and the def2-SVP basis set for *S* = 1/2. The data denotes the relative energies with added ZPE (kcal/mol). The structures on the *S* = 1/2 are shown in a 2D sketch. (B) Stationary point and transition structures associated with the oxidation of guaiacol in the active site of the WT (upper panel, porphyrin ring shown in green) and F169A P450_GcoA_ enzyme (lower panel, porphyrin ring shown in pink). A water molecule that is in the proximity of iron oxo in the F169A variant is shown by the stick mode. The key distances are shown in Å, and the angle of hydrogen atom abstraction is shown in degrees.

A previous study by Machovina et al. shows that F169A slightly enhanced activity toward the guaiacol,^20^ and our simulation shows that F169A mutation causes increased water inflow to the heme center. Hence, it must be elucidated whether water plays a role in catalysis. Therefore, we performed QM/MM calculations for the WT and F169A mutant to understand the enhanced activity. We started our QM/MM calculations with representative snapshots of WT and F169A mutant complexes taken from the most populated snapshots from the MD simulations and performed PES scanning of the QM/MM optimized structures. The reaction profiles and the reactive species for O-demethylation at methoxy carbon for the WT and F169A mutant (Figure 4) show that the oxidation of guaiacol is initiated by hydrogen atom abstraction (HAT) via Cpd I and goes through a transition state TS. This is in accordance with the previous DFT calculations.^19,20,27,28^ Interestingly, the methyl hydrogen of guaiacol in the WT complex is positioned at 2.12 Å and is closer to the oxo moiety of Cpd I than the methyl hydrogen of the mutant at 2.21 Å. However, the HAA barrier in the mutant (15.8 kcal/mol) is lower than the HAA barrier in the WT complex (19.4 kcal/mol). We found that *the additional water in the F169A mutant plays a pivotal role in stabilizing the transition state (at 1.18 Å) relative to the transition state (at 1.16 Å) in the WT complex*. The role of water near the Cpd I in stabilizing the transition state associated with HAA through electrostatic interactions is well documented by Shaik and co-workers.^11^ IM1 in both complexes are endothermic by a similar energy value and possess a similar geometry. Subsequently, the hydroxyl group is transferred to the substrate via a rebound mechanism and the hydroxylated product is formed in a barrierless process in both WT and mutant complexes. These results are in good agreement with the experimental data that the F169A mutant increases the guaiacol activity.^19,20^

To evaluate the effect of the aromatic triad consisting of F75, F169, and F395, which was suggested to help position the substrate in a catalytically competent orientation, we performed another set of QM/MM calculations using a different MD snapshot of WT P450_GcoA_ with the extended QM region to include the aromatic triad. The reaction barrier for HAA from Cpd I for this snapshot displayed a similar reaction barrier of 19.5 kcal/mol (Figure S4 and Table S2). The results are consistent with the QM/MM calculations with a smaller QM region, which indicates that the aromatic triad does not directly participate in the catalytic activity but determines substrate recognition and binding. Benchmarking was also done with the def2-TZVP basis set, which yielded a similar barrier to that obtained by def2-SVP. Therefore, the def2-SVP basis set was used in the subsequent calculations (Table S3).

### 3.3 Mechanism-Based Inhibition by the Singly Demethylated Intermediate of Syringol 3MC in WT CYP450_GcoA_

**Figure 5:**
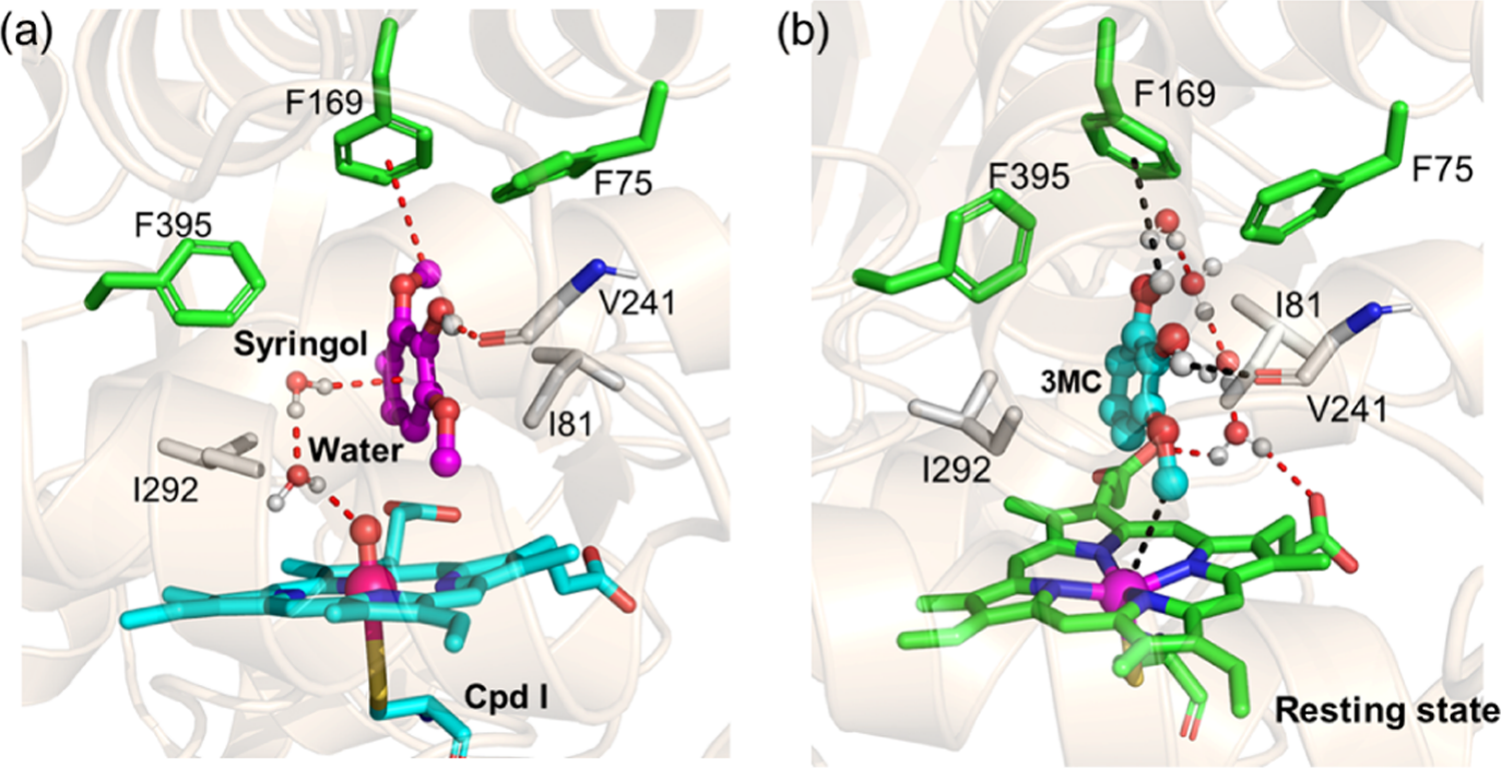
Active site of the WT P450_GcoA_ enzyme in a complex with (a) syringol in Cpd I and (b) 3MC intermediate in the resting state.

Syringol has an additional methoxy group in comparison to guaiacol, and it cannot be demethylated by the WT P450_GcoA_ enzyme.^20^ Interestingly, unlike the enzyme in the presence of guaiacol that displays mainly the closed conformation, the WT enzyme in the presence of syringol shows an open conformation of the substrate access channel during entire MD simulations (SI Figures S1 and S2) and is consistent with the previous literature.^19^ Therefore, we propose that *syringol may act as an allosteric modulator that triggers the channel opening in the WT P450*_*GcoA*_*enzyme*. To validate this allosteric effect, we carefully monitored the interaction of the syringol with nearby protein residues and found that the distal methoxy carbon of syringol favorably interacts with the π electron clouds of the F169 residue via C-H-π and C-H-O interactions (Figure 5a). Since F169 belongs to the substrate access channel, the interaction of syringol with F169 triggers the conformation of the substrate access channel to change from a closed to an open state. The open doorway allows water influx to access Cpd I (Figure 5a) and is in contrast with the previous simulations where no water molecules were found near Cpd I.^19,20^ However, similar to guaiacol, syringol also forms a strong hydrogen bond with the backbone oxygen of V241, which stabilizes syringol in the binding site.

MD simulations show that syringol induces open-channel conformation and binds tightly to the WT P450_GcoA_ enzyme. However, this creates a mechanistic enigma; if the syringol binds tightly and induces water access to the catalytic site, then how could it be correlated with the inactivity of syringol? It is worth noting that O-demethylation of syringol into the central intermediate occurs via two concomitant oxygen insertions on two methoxy groups of syringol (cf. Scheme 1c). After the first oxidation, intermediate 3MC needs to rotate to align its second methoxy group toward Fe (IV)=O of Cpd I for the second oxidation. This is only possible if syringol is able to move freely in the binding site. However, the MD simulations show that syringol binds very tightly to the WT enzyme and shows little mobility. Here, tight binding of syringol may inhibit further oxidation, and therefore, no activity is observed for S-unit in the WT P450_GcoA_ enzyme, which is similar to mechanism-based inhibition already been reported for cytochrome P450.^50^ To further validate this hypothesis, we performed a separate MD simulation of central intermediate 3MC in the resting state of WT CYP450_GcoA_. As per our expectations, intermediate 3MC was very stable in the resting state of the enzyme (see Figure 5b) and does not exhibit mobility and hence would account for inhibition of the second oxidation.

### 3.4 Mobility of 3MC Enables Successive Demethylation of Syringol in the F169A Mutant

**Figure 6:**
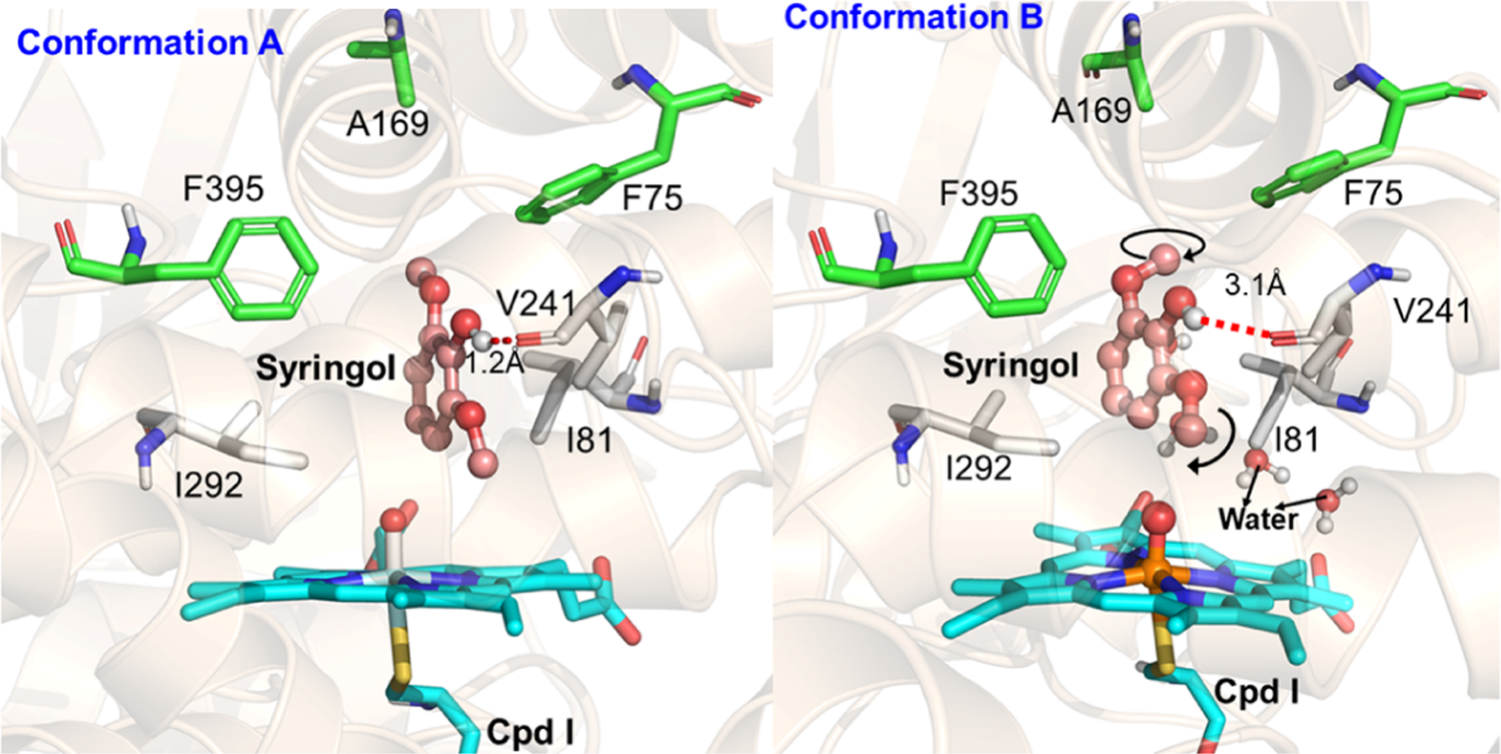
Two conformations of syringol were obtained from the MD simulations of the F169A mutant with syringol.

The study by Machovina et al. shows that the F169A mutant significantly affects the syringol activity, changing CYP450_GcoA_ from a specialized enzyme to a promiscuous one.^19^ We, therefore, simulated the F169A mutant in a complex with syringol. Interestingly, two conformations of the substrate (Figure 6) were observed, which are in contrast to the previous literature^19^ and indicate the importance of sampling in studying enzyme catalysis. In both conformations, one methoxy group was positioned proximal to Fe (IV)=O, while the other stays away at a large distance.

Unlike the WT complex with syringol, no C–H−π nor C–H–O interaction of the substrate with protein residues was observed due to the lack of the benzene ring in the mutant and the large distance between the substrate and the residue at position 169. Although the key active site residues maintain the same orientations in conformations A and B, the orientation of the substrate significantly differs in both conformations (see arrows in Figure 6). Due to the different orientations of syringol, it binds tightly with Val241 in conformation A, while it stays away in conformation B. Furthermore, a persistent water channel was found in conformation B that stretches between A169 and F395 residues and it solvates the substrate and the active site. Due to the lack of a stabilizing interaction in conformation B, the substrate shows a high degree of conformational mobility that may facilitate the second oxidation of intermediate 3MC.

**Figure 7:**
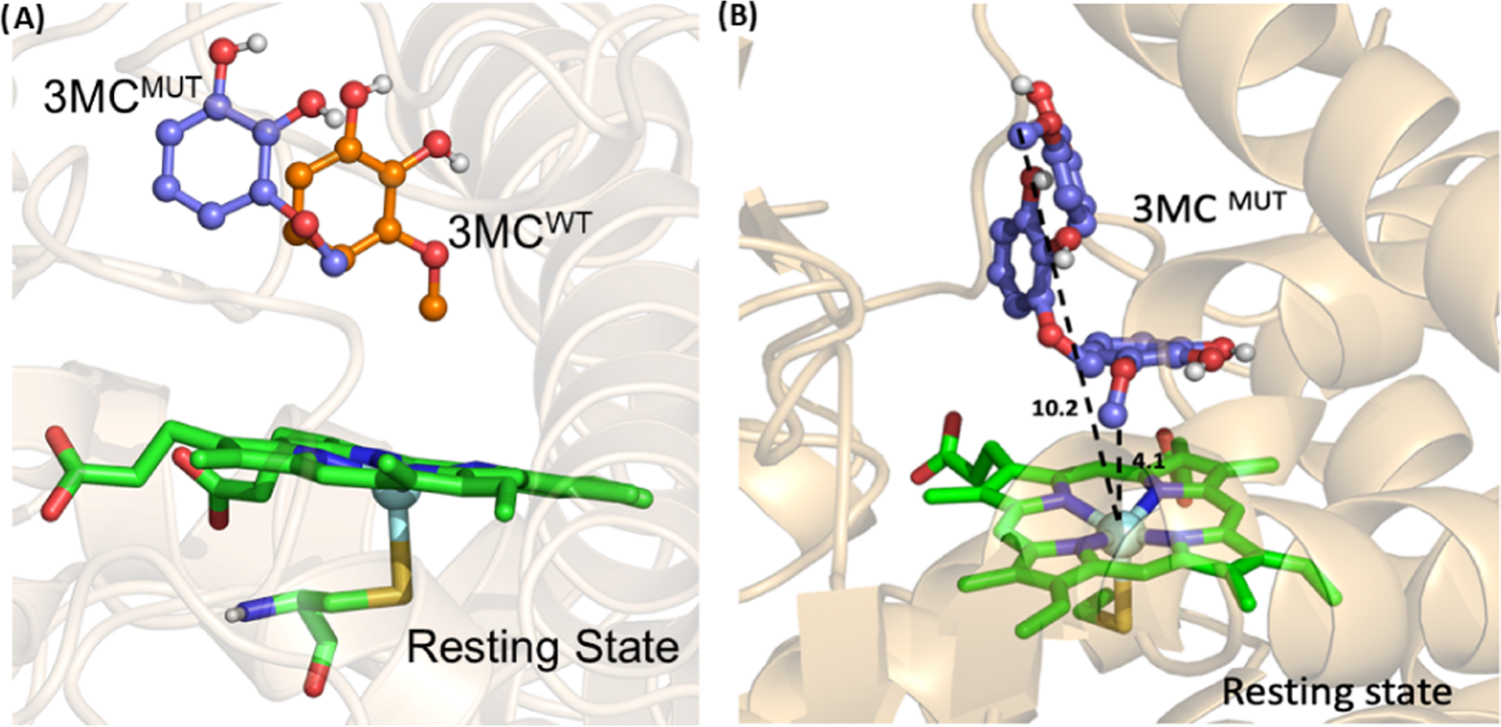
Comparison of the substrate (3MC) positioning in the WT and F169A mutant. Compared to the WT enzyme (A), the altered orientation of the substrate in the mutant provides extra space to reset the catalytic cycle for the second dose of O-demethylation. (B) Rearrangement of 3MC in the active site of the mutant from different intervals of the simulation.

As seen earlier, the 3MC intermediate was quite stable in the WT complex; therefore, we performed a separate MD simulation of 3MC in the resting state of the F169A mutant to study the behavior of 3MC in the mutant complex. Interestingly, the MD simulations reveal significant conformational mobility of 3MC in the resting state of the F169A mutant (Figure 7). The extra space created by F169A mutation allows 3MC to reorientate itself in the active site for the methoxy group to be positioned toward iron oxo as that second demethylation would take place to produce dual-demethylated product pyrogallol.

### 3.5 Mechanism of Aryl-*O*-demethylation in the F169A Mutant for Syringol

**Figure 8:**
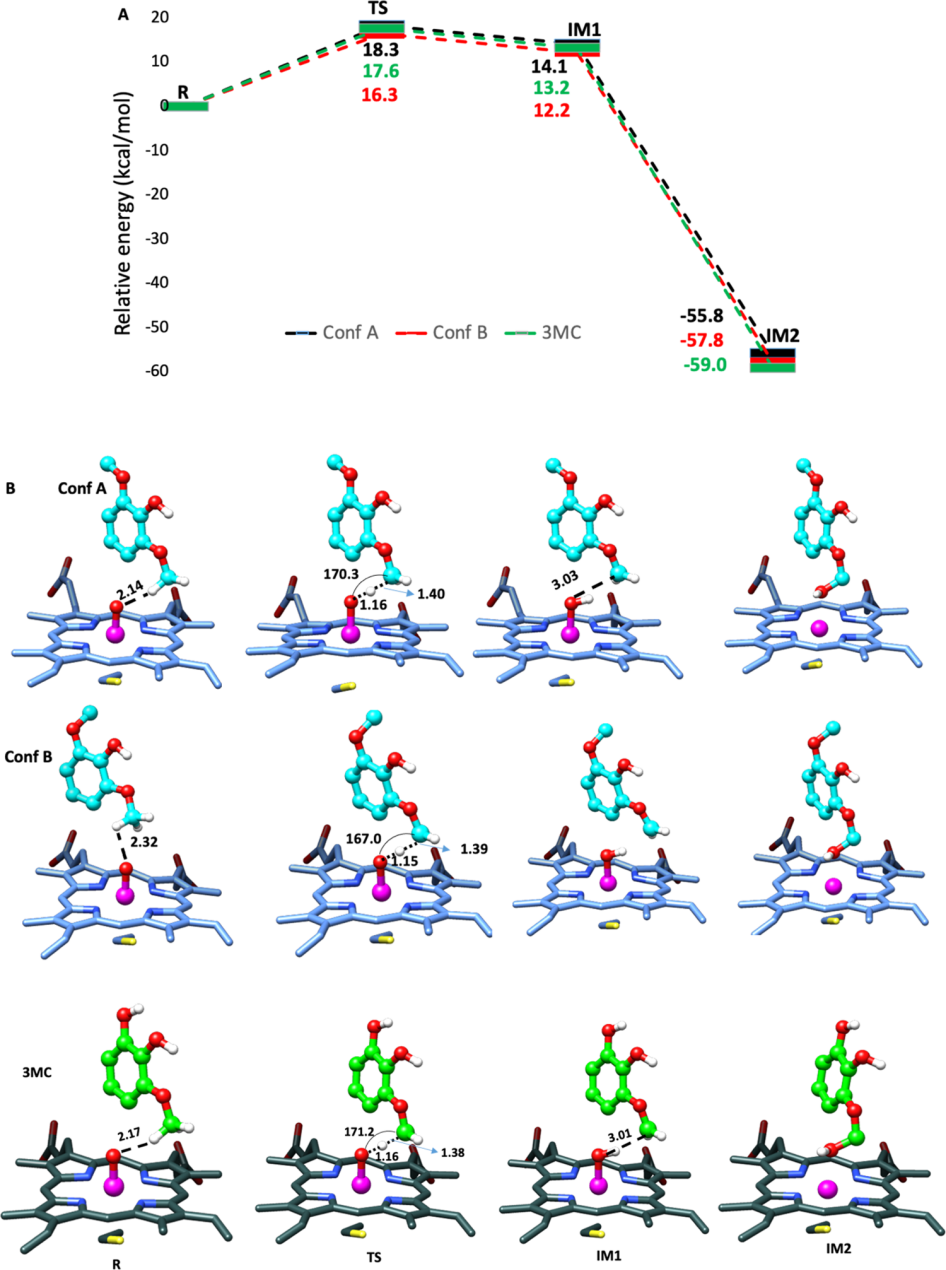
Oxidation of syringol in the active site of the F169A P450_GcoA_ enzyme. (A) QM/MM reaction profile was computed using the UB3LYP functional with D3BJ dispersion correction and the def2-SVP basis set for *S* = 1/2. The data denotes the relative energies with added ZPE (kcal/mol). (B) Stationary point and transition structures associated with the oxidation of syringol and 3MC in the active site of F169A-GcoA, in conformation A and conformation B. The key distances are shown in Å, and the angle of hydrogen atom abstraction is shown in degrees.

As shown in the previous section, syringol in the F169A mutant has two possible binding conformations: A and B. We, therefore, performed two separate QM/MM calculations to decipher how these alternate conformations contribute toward catalysis. Figure 8 shows the reaction profile for conformations A and B in red and black colors, respectively. In both conformations, aryl-*O*-demethylation occurs via HAA similar to Figure 3a and is consistent with the previous DFT studies on syringol oxidation.^19,27^ The TS barriers for the HAA process for both conformations are 18.3 and 16.3 kcal/mol, respectively, and the TS barrier of the HAA process in conformation B is lower in energy by 2.0 kcal/mol. Note that conformation A belongs to the conformation similar to WT, and therefore, we suggest that the F169A mutation causes enhanced flexibility; hence, the substrate adopts an alternative conformation (i.e., B) that is more reactive.

The experimental findings suggest that F169A performs two rounds of O-demethylation on the methoxy groups of syringol to yield pyrogallol.^19^Therefore, were performed QM/MM calculations for O-demethylation of 3MC using a similar protocol to that described above. We found that the TS barrier for HAA for the mutant is 17.6 kcal/mol, which is almost similar to the TS barrier for the HAA reaction in syringol as the substrate and follows a similar barrierless rebound step to the exothermic IM2 production (Figure 8).

## 4 Conclusions

Using extensive MD simulations and QM/MM calculations, we explored the mechanism of spontaneous promiscuity for the syringol activity due to F169A mutations in CYP450_GcoA_. Our study highlights some key aspects of a single-site mutation that has far-going consequences on the catalytic activity of P450GcoA as follows:

(a) The aromatic dyad of F169 and F395 forms a doorway that controls the water aqueduct that stretches to the heme site. Mutation of F169A opens the doorway and allows water influx to the heme site. QM/MM calculations show that the presence of water stabilizes the TS and hence lowers the reaction barrier, which in turn enhances the guaiacol activity in the mutant.
(b) In the WT enzyme, the S-lignin (syringol) binds very tightly even after substrate oxidation (3MC), which impedes the rotation of the substrate; therefore, the second O-demethylation of syringol is inhibited. F169A creates an extra room in the activity site, enabling the rearrangement of the 3MC intermediate and therefore facilitating the second O-demethylation of syringol.
(c) The study provides a peculiar mechanistic insight that the tight binding of the substrate (S-lignin) may have an inhibitory effect on the catalytic efficiency, and the conformational flexibility is of paramount importance in successive O-demethylation reactions. Therefore, experimental mutations in the active site binding residues such as V241, I81, and I292 in combination with F169A can possibly alter the flexibility of the GcoA active site to accommodate various 4-alkylguaiacols, hence making it even more promiscuous toward various downstream lignin byproducts.

Since P450GcoA catalyzes the downstream stage of lignin breakdown, which is of commercial importance, our understanding of the enzyme promiscuity due to the presence of water channel and substrate mobility shed light on further bioengineering study of degradation of analogous lignin feedstock.

## Supporting Information Available

Coordinates for the QM region for different reactive species, root-mean-square deviations, root-mean-square fluctuations, and so on (PDF)
