# Supplementary material for: Single-Site Mutation Induces Water-Mediated Promiscuity in Lignin Breaking Cytochrome P450GcoA
Source: ACS Omega. 2022 Jun 10;7(24):21109–18. doi: 10.1021/acsomega.2c00524 (PMC9219061; doi:10.1021/acsomega.2c00524)
Supplement: Supplementary file 1 — ao2c00524_si_001.pdf [file ao2c00524_si_001.pdf]

Supporting information

A Single Site Mutation Induces a Water-Mediated Promiscuity in Lignin Breaking Cytochrome P450GcoA

Warispreet Singh* [1,2], Sónia F. G. Santos [1,2], Paul James [1,2], Gary W Black [1,2] Meilan Huang*[3], Kshatresh Dutta Dubey [4]*

[1] Department of Applied Sciences, Northumbria University, Newcastle upon Tyne, NE1 8ST, United Kingdom

[2] Hub for Biotechnology in Build Environment, Newcastle upon Tyne, United Kingdom

[3] Department of Chemistry & Chemical Engineering, Queen's University, Belfast, BT9 5AG, United Kingdom

[4] Department of Chemistry and Centre for Informatics, Shiv Nadar University Delhi NCR, 201314, India.

Corresponding Authors: w.singh@northumbria.ac.uk, m.huang@qub.ac.uk kshatresh@gmail.com

Table of Contents

Table S1 The MD setups used in this study……………………………………………………………..S4
Table S2 The QM/MM energies for the WT $P450_{GcoA}$ enzyme in complex with guaiacol with additional QM region. S4
Table S3 The QM/MM energies for the F169A $P450_{GcoA}$ enzyme in complex with guaiacol using def2-SVP and def2-TZVP basis sets for an additional snapshot obtained from the equilibrated MD trajectory............... S5
Scheme 1 The QM region used in the QM/MM calculations S6
Figure S1-S4 The RMSD of Cα atoms of the different enzyme substrate complexes S7-S10
Figure S5. The opening and closing of the channel ………………………………………………………S11
Figure S6 The radial distribution function of WT $P450_{GcoA}$ and F169A mutant in complex with guaiacol or syringol for 500ns trajectories... S12
Figure S7. The stationary point and transition structures associated with the oxidation of guaiacol in the active site of WT P450GcoA enzyme. S13
Figure S8 The distance plot as the function of time for WT and F169A P450GocA in complex with guaiacol and syringol.. S14
Figure S9 The centre of mass distance of F169 residue to the methoxy group of the syringol in wild type syringol complex. ……………………………………………………………………………………S15
References ……………………………………………………………………………………………S16

Docking of 3-methoxycatechol

The flexible ligand docking of 3-Methoxycatechol (3MC) was performed using the X-ray structures of F169A-syringol (PDB: 6HQQ[1]) and Wt-GcoA (PDB: 6HQK[1]) using AutoDock 4.2[2] suite with the Lamarckian genetic algorithm (LGA) and the standard free energy scoring function for both the Ferric penta-coordinate high-spin resting and the CpdI state. The grid box was centred at the oxygen atom of Fe (IV)=O motif of CpdI (X,Y,Z: 50.301Å x 44.243 Å x 50.171Å). In the case of resting state, the grid box was centred at the iron atom of the heme group (X,Y,Z: 49.597Å x 45.571Å x 50.827Å). The partial charges during the docking was obtained from the previous literature[3] and total of of 300 LGA runs were carried out for each ligand: protein complex.

Table S1 The MD setups used in this study

S.No	Name	PDB code	Resolution (Å)
1	Wt-GcoA - guaiacol	6HQK[1]	1.57
2	F169A - guaiacol	5NCB[4]	1.44
3	Wt-GcoA - syringol	5OMU[4]	1.95
4	F169A- Ssyringol	6HQQ[1]	1.66
5	* F169A- 3MC	6HQQ[1]	1.66
6	F169A- 3MC	6HQQ[1]	1.66
7	*Wt-GcoA - 3MC	6HQK[1]	1.57

* GcoA-F169A-3MC Ferric penta-coordinate high-spin resting state and

*Wt-GcoA Ferric penta-coordinate high-spin resting state, 3-methoxycatechol (3MC)

Table S2 The QM/MM energies for the WT $P450_{GcoA}$ enzyme in complex with guaiacol with additional QM region.

	E	ZPE	E+ZPE	E kcal/mol	E +ZPE kcal/mol
R	-4920.91235	0.70453569	-4920.207814	0	0
TS	-4920.87558	0.69892456	-4920.176656	23.0728136	19.5517849
IM1	-4920.8863	0.70089929	-4920.185401	16.346241	14.0643681

Table S3 The QM/MM energies for the F169A $P450_{GcoA}$ enzyme in complex with guaiacol using def2-SVP and def2-TZVP basis sets for an additional snapshot obtained from the equilibrated MD trajectory

	E kcal/mol SVP	E+ZPE kcal/mol SVP	E kcal/mol TZVP	E+ZPE kcal/mol TZVP
R	0	0	0	0
TS	20.2751566	16.2018414	20.5402521	16.4669369
IM1	11.64852538	9.58738886	17.0751474	15.0140109
P	-58.75426354	-56.6066031	-51.627048	-49.4793875

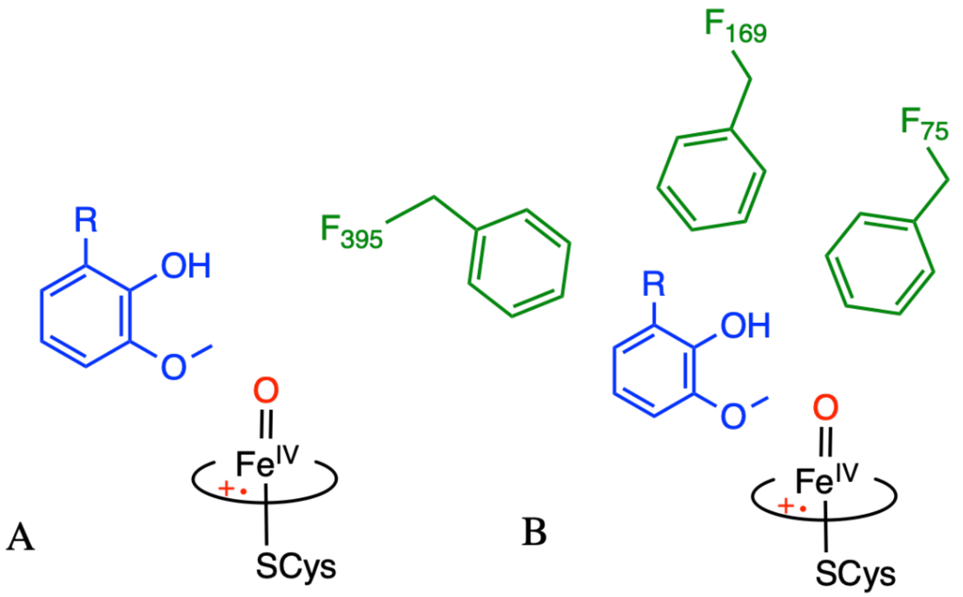

Scheme 1 The QM region used in the QM/MM calculations. (A) Cpd I and substrate are included in the QM regions to study the hydrogen atom abstraction and the rebound step. (B) Cpd I and substrate and F75, F169 and F395 were included in the QM region to study the hydrogen atom abstraction step. (R=H, O-CH_3, OH)

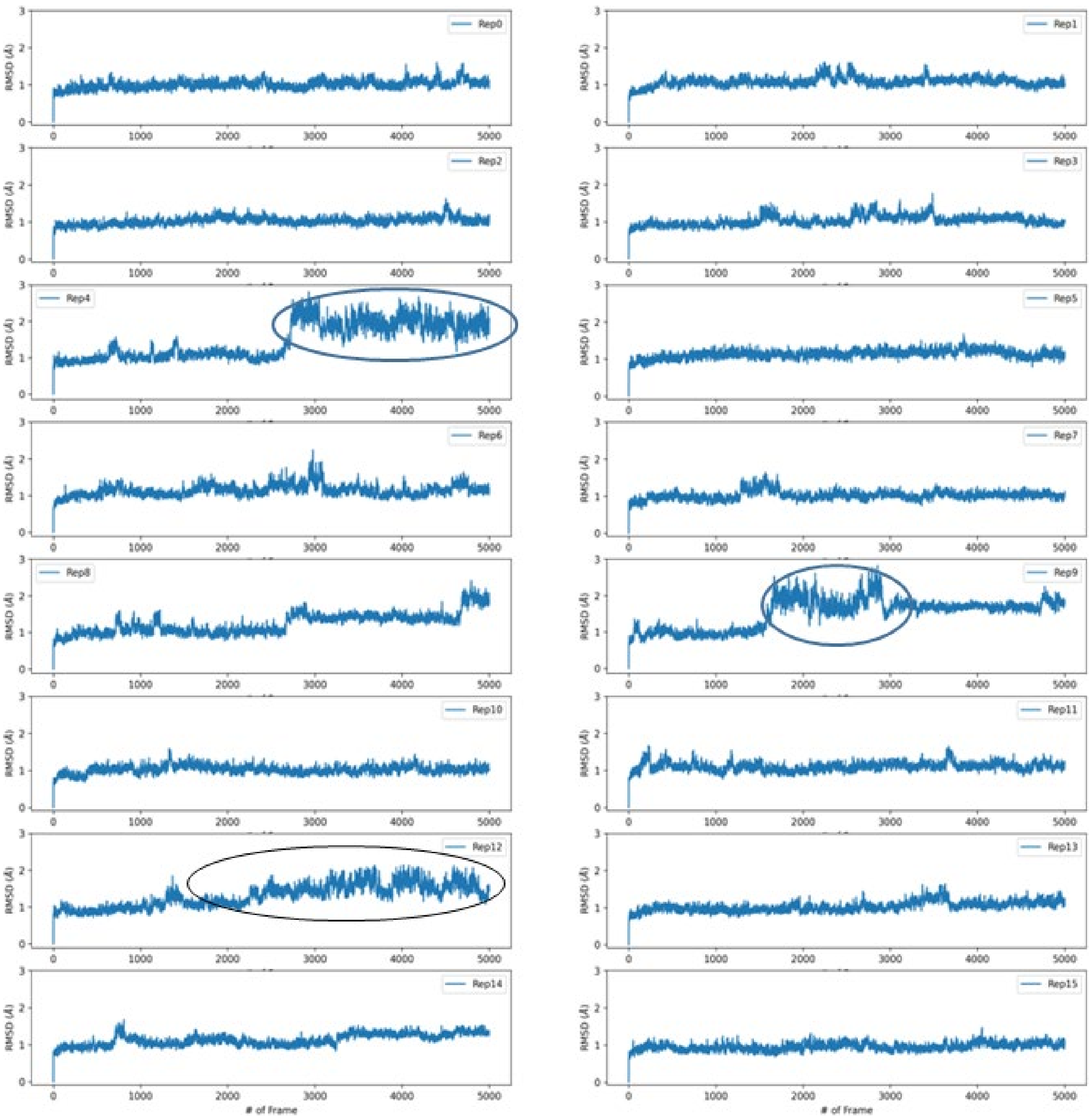

Figure S1. The RMSD of the Cα carbon Guaiacol and WT enzyme complex in Cpd I state. The encircled region highlights the flexibility.

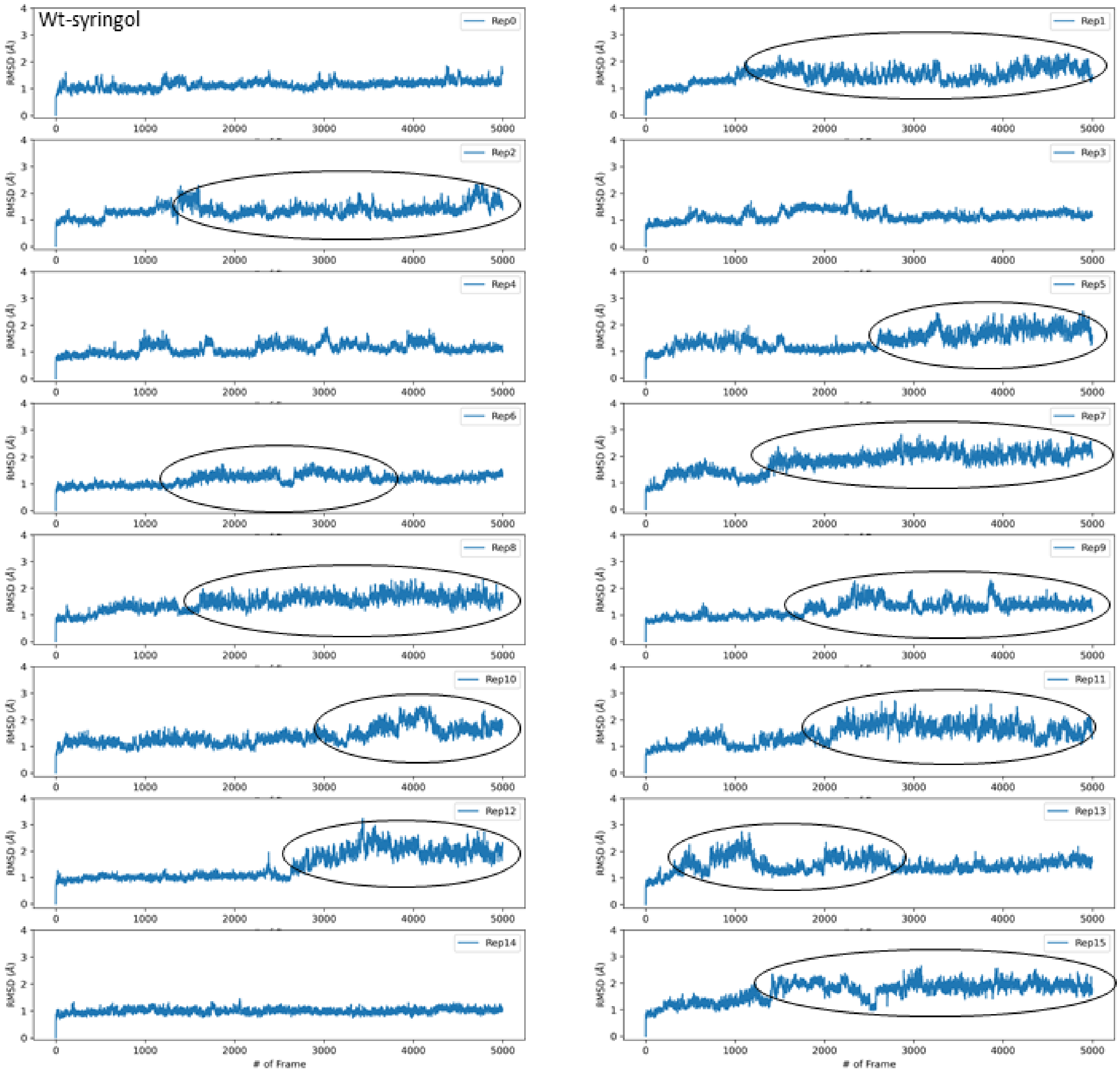

Figure S2. The RMSD of the Cα carbon Syringol and WT enzyme complex in Cpd I state. The encircled region highlights the flexibility.

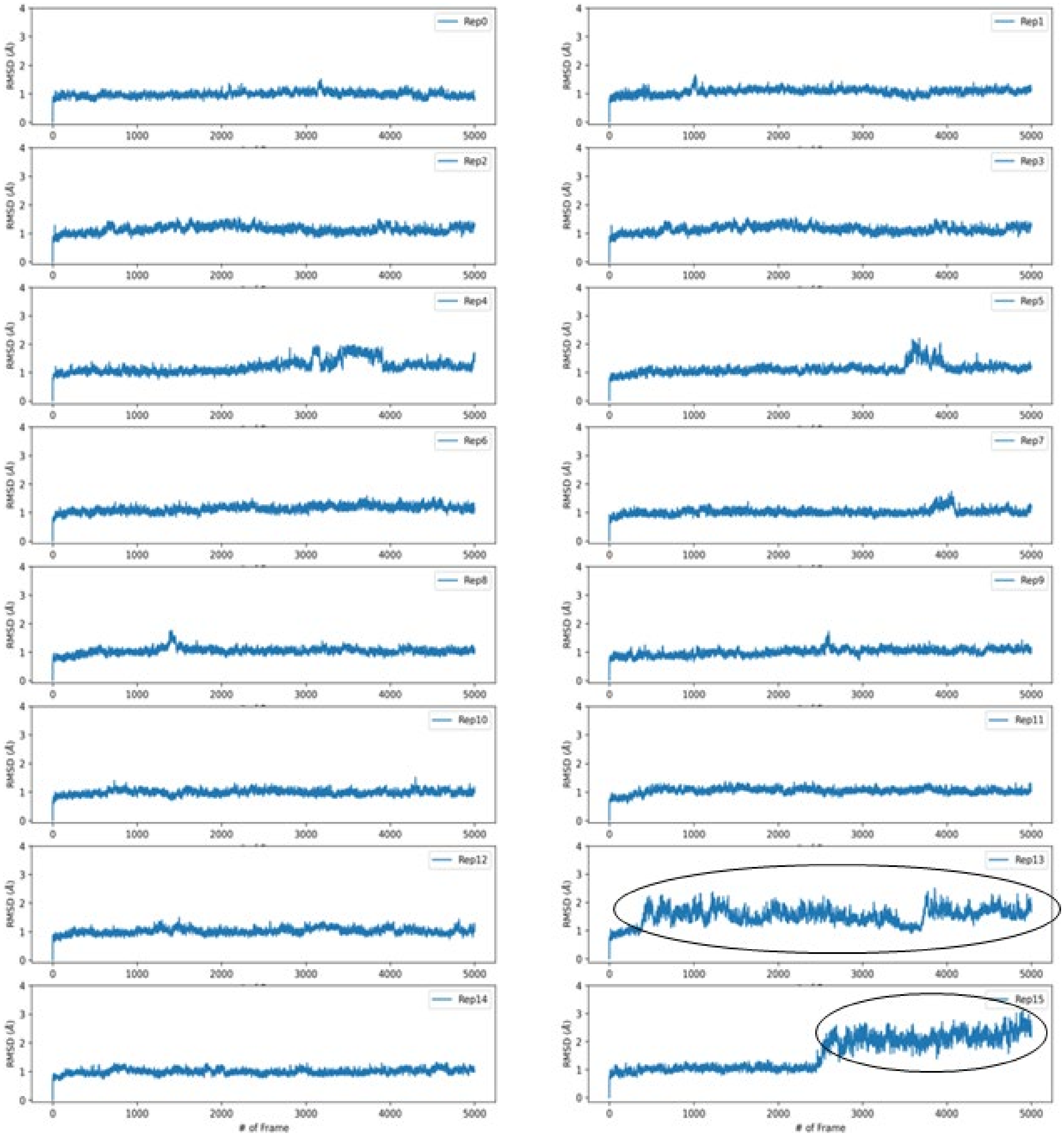

Figure S3. The RMSD of the Cα carbon Guaiacol and F169A MT enzyme complex in Cpd I state. The encircled region highlights the flexibility.

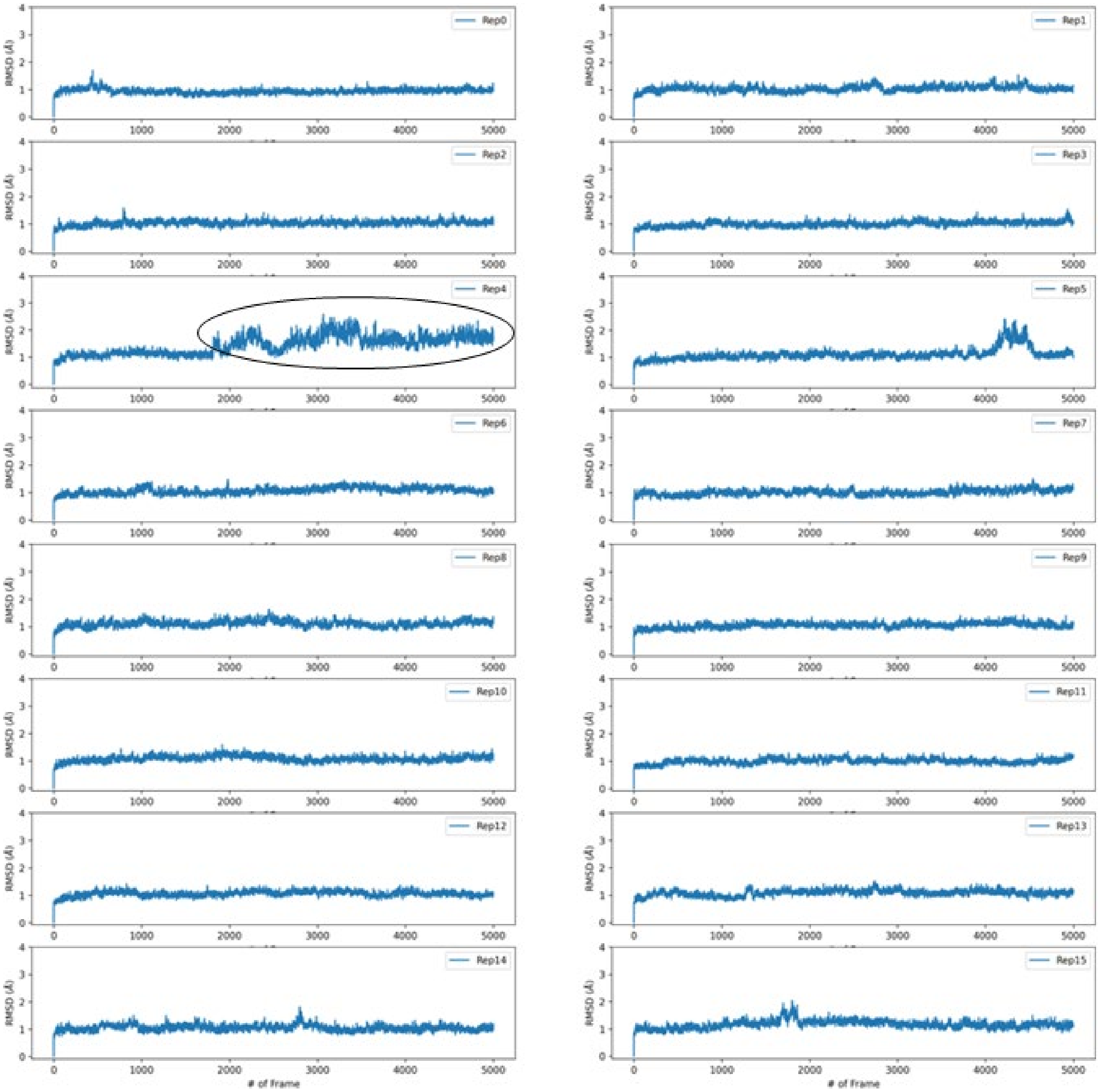

Figure S4. The RMSD of the Cα carbon Syringol and F169A MT enzyme complex in Cpd I state. The encircled region highlights the flexibility.

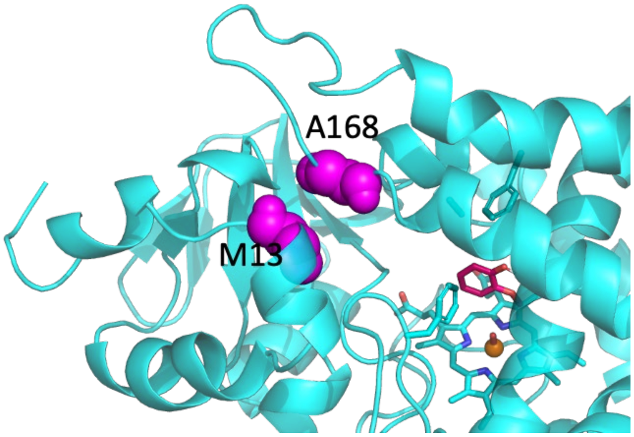

Figure S5 The opening of the substrate access loop located between F and G helices. The distance between the C-alpha atom of the M13 and A168 residue were measured as a function of time. The red line represents the distance of these residues in the X-ray structure.

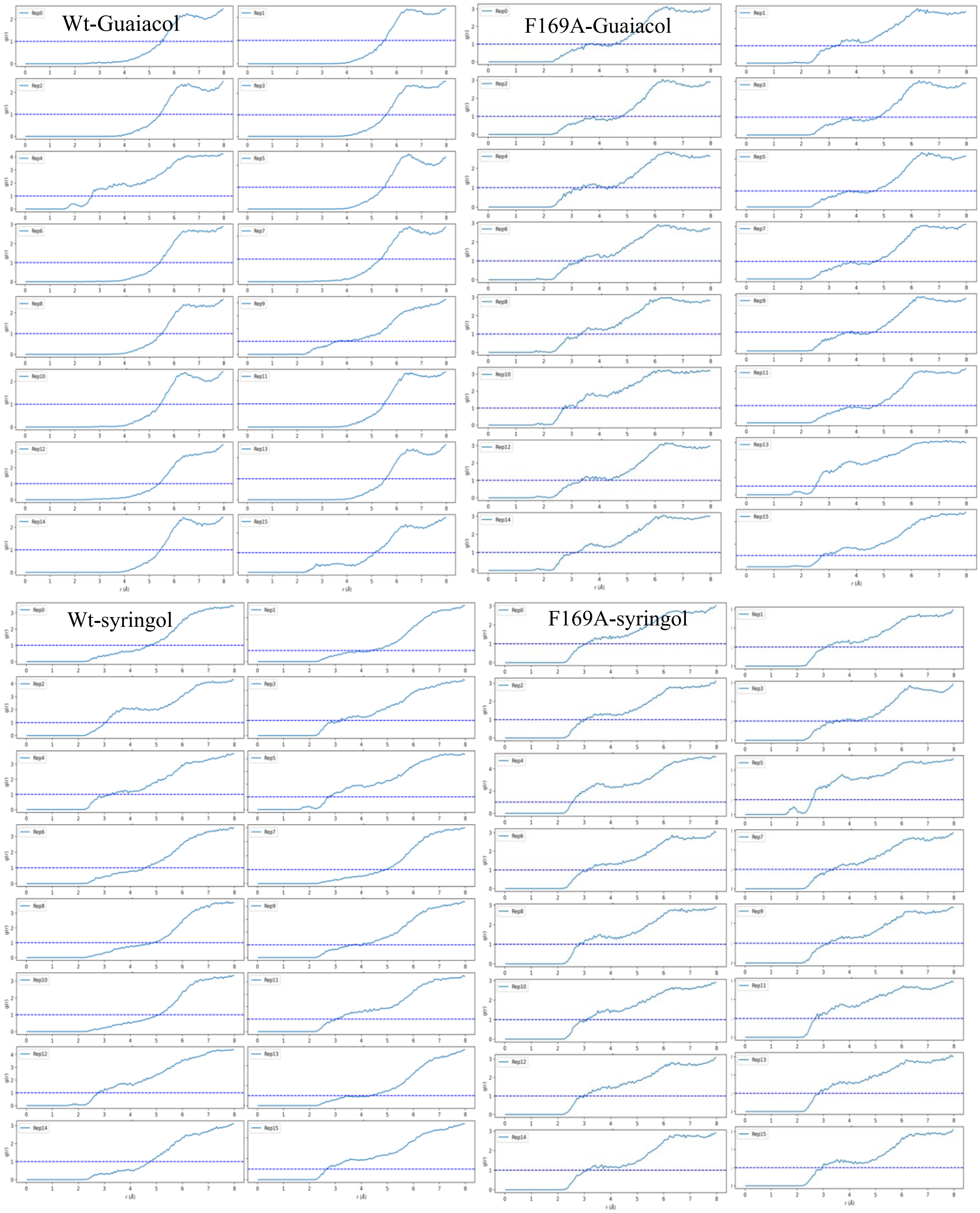

Figure S6 The radial distribution function of WT P450 $_{GcoA}$ and F169A mutant in complex with guaiacol or syringol for 500ns trajectories

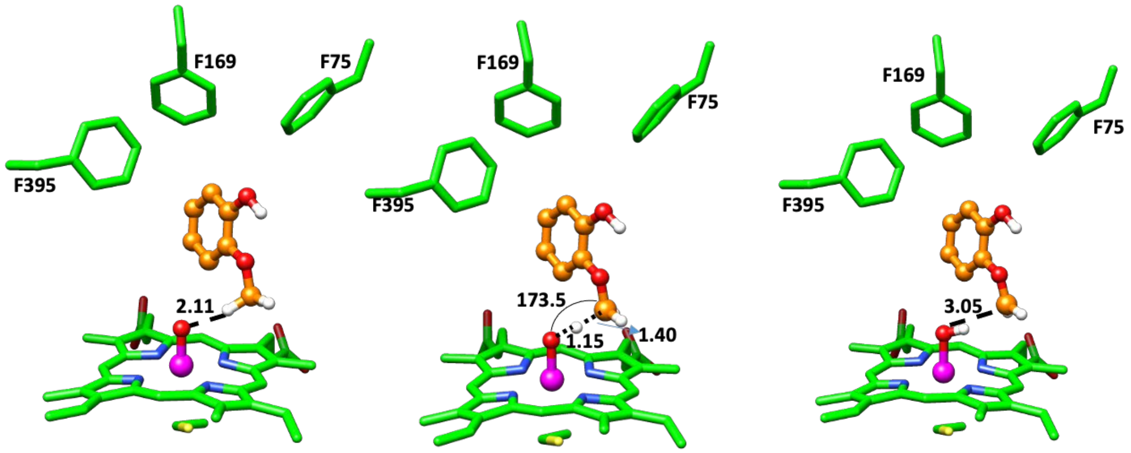

Figure S7. The stationary point and transition structures associated with the oxidation of guaiacol in the active site of WT P450GcoA enzyme. The key distances are shown in (Å), angle of hydrogen atom abstraction is shown in degree.

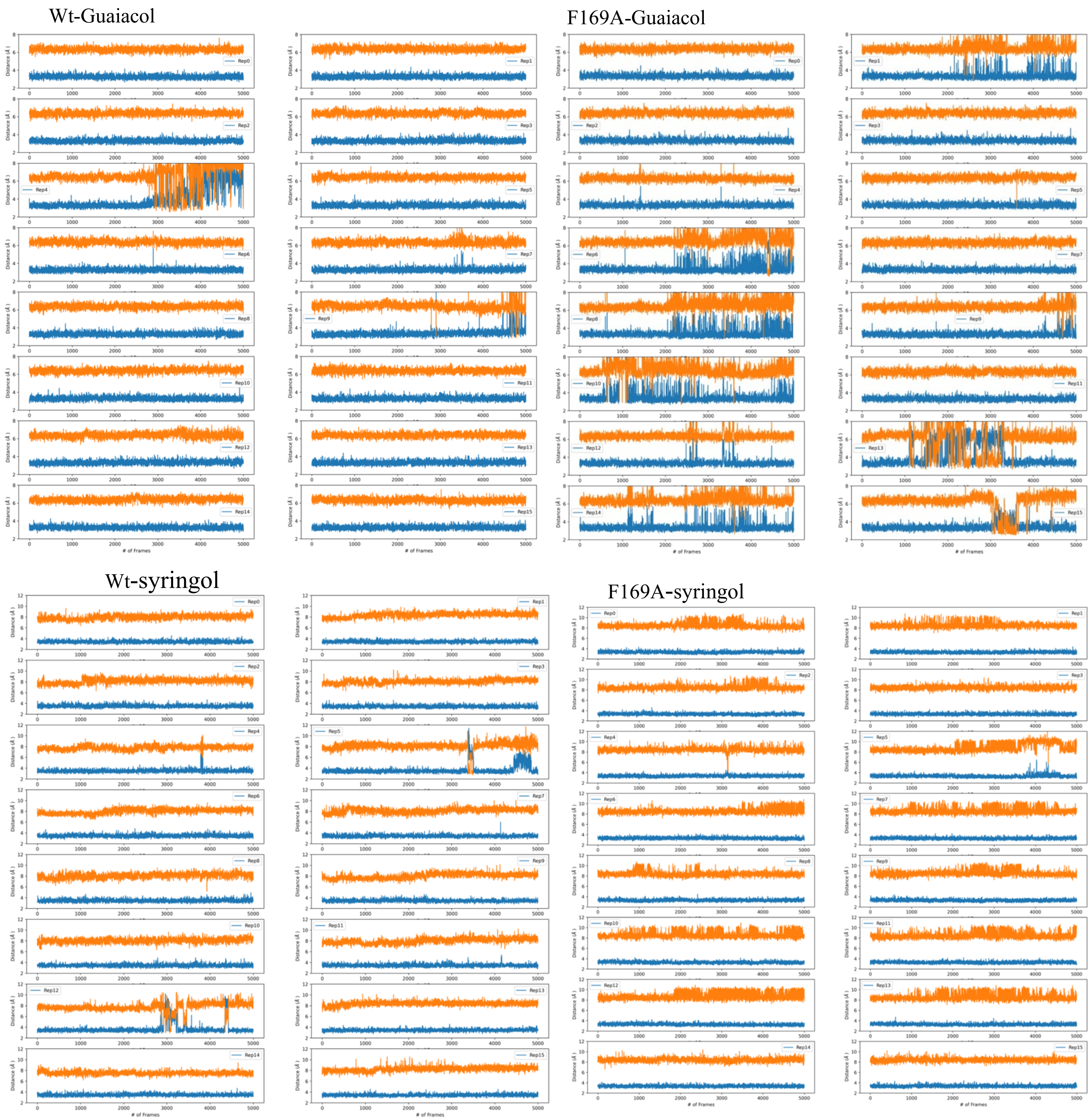

Figure S8 The distance plot as the function of time for WT and F169A P450GocA in complex with guaiacol and syringol. The methoxy (blue) and hydroxy group (orange) of guaiacol from the oxygen atom of Fe(IV)=O complex in WT and F169A P450 $_{GocA}$. In the case of syringol the distance of other methoxy group instead of hydroxyl group is measured.

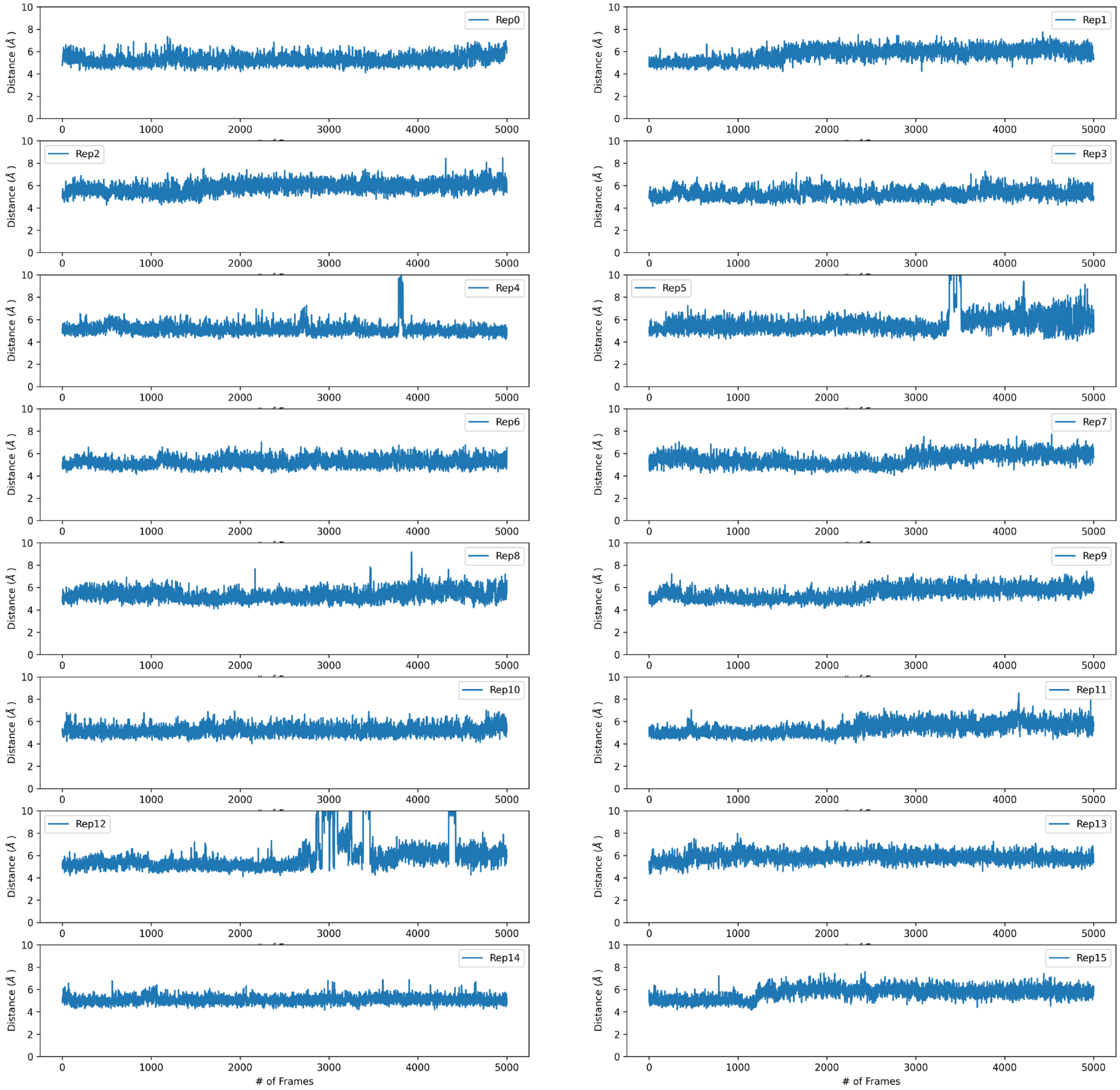

Figure S9 The centre of mass distance of F169 residue to the methoxy group of the syringiol in wild type syingiol complex.

References

Machovina, M. M.; Mallinson, S. J. B.; Knott, B. C.; Meyers, A. W.; Garcia-Borràs, M.; Bu, L.; Gado, J. E.; Oliver, A.; Schmidt, G. P.; Hinchen, D. J.; Crowley, M. F.; Johnson, C. W.; Neidle, E. L.; Payne, C. M.; Houk, K. N.; Beckham, G. T.; McGeehan, J. E.; Dubois, J. L., Enabling microbial syringol conversion through structure-guided protein engineering. *PNAS.* **2019,** *116* (28), 13970-13976.

2. Mallinson, S. J. B.; Machovina, M. M.; Silveira, R. L.; Garcia-Borràs, M.; Gallup, N.; Johnson, C. W.; Allen, M. D.; Skaf, M. S.; Crowley, M. F.; Neidle, E. L.; Houk, K. N.; Beckham, G. T.; Dubois, J. L.; McGeehan, J. E., A promiscuous cytochrome P450 aromatic O-demethylase for lignin bioconversion. *Nat. Commun* **2018,** *9* (1).

3. Morris, G. M.; Huey, R.; Lindstrom, W.; Sanner, M. F.; Belew, R. K.; Goodsell, D. S.; Olson, A. J., AutoDock4 and AutoDockTools4: Automated docking with selective receptor flexibility. *J.Comput. Chem.* **2009,** *30* (16), 2785-2791.

4. Shahrokh, K.; Orendt, A.; Yost, G. S.; Cheatham, T. E., Quantum mechanically derived AMBER-compatible heme parameters for various states of the cytochrome P450 catalytic cycle. *J.Comput. Chem.* **2012,** *33* (2), 119-133.
